# Lipophosphoglycans from dermotropic *Leishmania infantum* are more pro-inflammatory than those from viscerotropic strains

**DOI:** 10.1590/0074-02760200140

**Published:** 2020-09-21

**Authors:** Camila A Cardoso, Gabriela V Araujo, Carmen M Sandoval, Paula M Nogueira, Concepcion Zúniga, Wilfredo H Sosa-Ochoa, Márcia D Laurenti, Rodrigo P Soares

**Affiliations:** 1 Fundação Oswaldo Cruz-Fiocruz, Instituto René Rachou, Belo Horizonte, MG, Brasil; 2 Universidade de São Paulo, Faculdade de Medicina, Departamento de Patologia, Laboratório de Patologia de Moléstias Infecciosas, São Paulo, SP, Brasil; 3 Hospital Escuela Universitario, Departamento de Vigilancia de la Salud, Tegucigalpa, Honduras; 4 Universidad Nacional Autónoma de Honduras, Instituto de Investigación en Microbiología, Tegucigalpa, Honduras

**Keywords:** lipophosphoglycan, Leishmania infantum, innate immunity

## Abstract

Although *Leishmania infantum* is well-known as the aethiological agent of visceral leishmaniasis (VL), in some Central American countries it may cause atypical non-ulcerated cutaneous leishmaniasis (NUCL). However, the mechanisms favoring its establishment in the skin are still unknown. Lipophosphoglycan (LPG) is the major *Leishmania* multivirulence factor involved in parasite-host interaction. In the case of viscerotropic *L. infantum*, it causes an immunosuppression during the interaction with macrophages. Here, we investigated the biochemical and functional roles of LPGs from four dermotropic *L. infantum* strains from Honduras during *in vitro* interaction with murine macrophages. LPGs were extracted, purified and their repeat units analysed. They did not have side chains consisting of Gal(β1,4)Man(α1)-PO_4_ common to all LPGs. Peritoneal macrophages from BALB/c and C57BL/6 were exposed to LPG for nitric oxide (NO) and cytokine (TNF-α and, IL-6) production. LPGs from dermotropic strains from Honduras triggered higher NO and cytokine levels compared to those from viscerotropic strains. In conclusion, LPGs from dermotropic strains are devoid of side-chains and exhibit high pro-inflammatory activity.

*Leishmania infantum* is well known as the cause of visceral leishmaniasis (VL) in the New World. In Central America, it has been shown that *L. infantum* also causes cutaneous lesions known as atypical cutaneous leishmaniasis (ACL) or non-ulcerated cutaneous leishmaniasis (NUCL). Early studies have already detected these forms in transmission areas in Costa Rica, El Salvador, Honduras and Nicaragua. In Honduras, parasitological examination of those strains from vertebrate and invertebrate hosts using isoenzymes allowed their identification as *Leishmania donovani chagasi* (nowadays *L. infantum*).^1^ ^,^ ^2^ ^,^ ^3^ ^,^ ^4^ An interesting feature of NUCL is that in ACL patients no indication of prior visceralisation occurred and they were not immunocompromised and/or malnourished.^5^ NUCL is a benign form affecting children and human immunodeficiency virus (HIV)-patients in Europe and their role as hosts in those endemic areas should be considered by public health authorities.^6^ Most of the mechanisms underlying persistence of a viscerotropic species in the skin is still unknown. This is different from viscerotropic species *L. donovani*, where egested microbiota and IL-1β production are crucial for parasite migration from skin to organs.^7^

In this context, several studies have assessed dermotropic *L. infantum* strains to understand their behavior in the skin. Macroscopically, NUCL is characterised by the presence of a small (0.1-3 cm) non-ulcerative erythematous papules surrounded by a hypopigmented halo on the exposed body areas including the face and extremities.^1^ Recently, the immunopathological features of the lesions from Honduran strains were microscopically described.^8^ ^,^ ^9^ Alike most dermotropic *Leishmania* species,^10^ ^,^ ^11^ the pro-inflammatory infiltrated in the dermis consisted of mononuclear cells including lymphocytes, macrophages and a few plasma cells. An interesting feature of the lesions was the scarcity of parasites even if the infiltrates were discreet or intense.^8^ Assessment of the local regulatory immune response in these lesions have detected the participation of FoxP3+ cells and TGF-β.^9^ Altogether these studies suggest that a regulatory cellular immune response could promote low parasite persistence and tissue damage by ACL strains. Thus, these mechanisms could hinder parasite migration to organs as opposed to *L. donovani* IL-1β-induced inflammasome.^7^ However, the role of parasite virulence factors in this process remains unknown.

Glycoconjugates of parasitic protozoans play a pivotal role during the parasite-host interaction. In *Leishmania*, lipophosphoglycan (LPG) is a multivirulence factor expressed on promastigote surface. LPG has four motifs: (i) a conserved glycan core region of 1-*O*-alkyl-2-lyso-phosphatidylnositol (PI); (ii) a core composed of Gal(α1,6)Gal(α1,3)Gal_f_(β1,3)[Glc(α,1)-PO_4_]Man(α1,3)Man(α1,4)-GlcN(α1) heptasaccharide; (iii) a portion of phosphorylated repeat units Gal(β1,4) Man(α1)-PO_4_; and (iv), a terminal neutral oligosaccharide (cap).^12^ *Leishmania infantum* LPG structures have been biochemically characterised in several strains from Brazil, Europe and Africa. Most of the strains possess type I LPG, whose repeat units are devoid of side chains. Only 10% of strains are branched-off with 1-3 β-glucose side-chains indicating the LPG polymorphisms are very low for *L. infantum*.^13^ ^,^ ^14^ Several functions have been elucidated for *L. infantum* LPG during interaction with macrophages and other immune cells. These include: TLR2/TLR4 agonists, NF-kB translocation, induction of heme-oxygenase-1 and prostaglandin E_2_.^15^ ^,^ ^16^ ^,^ ^17^ Compared to other dermotropic *Leishmania* species, *L. infantum* LPG exhibit a more immunosuppressive behavior, whereas *L. braziliensis* and *L. amazonensis* are pro-inflammatory.^15^ ^,^ ^18^ Glycobiology studies on *L. infantum* has focused only on viscerotropic strains from humans and dogs and no information on LPG structures from dermotropic Central America strains is available.

As part of a wider study on *L. infantum* glycobiology,^13^ ^,^ ^14^ this work purified and characterised the repeat units from dermotropic *L. infantum* causing NUCL in Honduras. Additionally, the activity of purified LPGs from dermotropic and viscerotropic was evaluated in murine macrophages.

All strains used in this study were from the Biorepository of Laboratorio de Patologias Infecciosas at University of São Paulo (USP). They were originally isolated from patients with NUCL from Amapala (Honduras)^8^ ^,^ ^9^ and included: MHOM/HN/2017/AM-65, MHOM/HN/2017/AM-73, MHOM/HN/2018/AMA-161 and MHOM/HN/2018/AMA-614. Also, for functional macrophage studies, Brazilian viscerotropic strains were included (MHOM/BR/1970/BH46 and MCAN/BR/89/BA262).^14^ Promastigotes were grown in M199 (Sigma, St. Louis, MO) and LPGs were extracted and purified from early stationary phase using a solvent E (H_2_O/ethanol/diethyl ether/pyridine/NH_4_OH; 15:15:5:1:0.017) (All from Merck, Darmstadt, Germany) as previously reported.^13^ ^,^ ^14^ To confirm purification, LPGs were resolved in sodium dodecyl sulphate-polyacrylamide gel electrophoresis (SDS-PAGE) (Bio-rad, Berkeley, CA) and transferred to nitrocellulose paper (Bio-rad). The membrane was blocked for 1 h in 5% milk (Molico, Vevey, Vaud) in phosphate-buffered saline (PBS) and probed overnight with monoclonal antibody (mAb) CA7AE (1:1,000), which recognises the unsubstituted Gal(β1,4)Man repeat units.^19^ After three washes in PBS, the membrane was incubated for 1 h with anti-mouse IgG conjugated with peroxidase (1:10,000) (Sigma) and the reaction was visualized using luminol (Bio-rad) (Fig. 1A). The LPGs from all dermotropic *L. infantum* strains were recognised by the mAb CA7AE, allowing the visualisation of characteristic smears common to all LPGs.^13^ ^,^ ^14^ These results indicate that some repeat units were indeed unsubstituted. To confirm this, purified LPGs were subjected to mild acid hydrolysis (0.02 N HCl, 5 min, 100ºC) (Sigma) to depolymerize the repeat units.^13^ Water-soluble fractions were partitioned using 1-butanol (Merck) and repeat units were treated with alkaline phosphatase (15 mM Tris buffer, pH 9,0, 1 U, 16 h, 37ºC) (Sigma). The neutral repeat units were desalted by passage through a two-layered column of AG50W-X12 (H^+^) over AG1-X8 (acetate) (Bio-rad). Then, samples were fluorescently labeled with 0.05 N ANTS (8-aminonaphthalene-1,3,6-trisulfate) and 1 M cyanoborohydride (37ºC, 16 h) (Sigma). They were subjected to fluorophore-assisted carbohydrate electrophoresis (FACE) using oligo-glucose ladders (G_1_-G_7_)^14^ as standards (Sigma) (Fig. 1B). All strains exhibited only one band co-migrating with the standard oligo-glucose ladder Glc_2_ indicating the presence of the disaccharide Gal(β1,4) Man(α1). This profile is consistent with type I LPG, which was observed for 90% of the viscerotropic *L. infantum* strains.^14^ Also, these LPGs were very similar to that from *L. donovani* (Sudan) and dermotropic species including *L. braziliensis*, *Leishmania shawi* and *Leishmania enriettii*.^20^ ^,^ ^21^ ^,^ ^22^ ^,^ ^23^ Regardless of the tropism, LPGs from Old and New World *L. infantum* strains are devoid of side-chains reinforcing that this glycoconjugate has low polymorphism in this species. The qualitative information on LPG polymorphisms in the repeat units of *L. infantum* and *L. donovani* are summarised in Table.

**Fig. 1:**
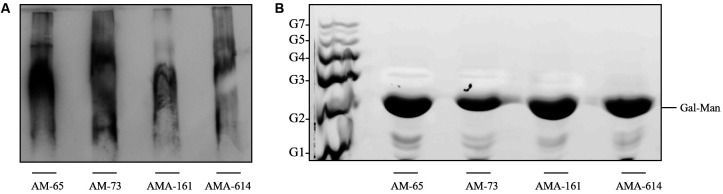
analysis of the lipophosphoglycans (LPGs) from dermotropic *Leishmania infantum* strains (AM-65, AM-73, AMA-161 and AMA-614): (A) Immunoblotting of purified intact LPG from promastigotes of *L. infantum* strains probed with mAb CA7AE (1:1,000). (B) Fluorophore-assisted carbohydrate electrophoresis (FACE) of LPG repeat units. Lane 1, oligo-glucose ladder represented by G_1_-G_7_; lanes 2-5, repeat units of AM-65, AM-73, AMA-161 and AMA-614 strains, respectively.

**TABLE:** Update on lipophosphoglycan (LPG) structures of *Leishmania donovani* complex species from Old and New World countries

Species/strains^*a*^	Clinical pattern^*b*^	Origin (city/state^*c*^ /country)	LPG type	Ref.
*Leishmania infantum*				
MCAN/BR/89/Ba-262	CanL	Jacobina/BA/Brazil	I	^(14)^
MHOM/BR/2001/HP-EMO	VL	Pancas/ES/Brazil	I	^(14)^
MHOM/BR/1987/HCO-1	VL	ND/ES/Brazil	I	^(14)^
MCAN/BR/99/JP15	CanL	João Pessoa/PB/Brazil	I	^(14)^
MHOM/BR/1985/GS	VL	ND/BA/Brazil	I	^(14)^
MHOM/BR/2003/MMF	VL	Cipolândia/MS/Brazil	I	^(14)^
240 (dog/BR/ND)	CanL	Belo Horizonte/MG/Brazil	I	^(14)^
291 (ND/BR/ND)	ND	Aracaju/SE/Brazil	I	^(14)^
MCAN/BR/2004/CUR268	CanL	Belo Horizonte/MG/Brazil	I	^(14)^
MCAN/BR/2004/CUR269	CanL	Belo Horizonte/MG/Brazil	I	^(14)^
MCAN/BR/2003/CUR211	CanL	Belo Horizonte/MG/Brazil	I	^(14)^
MCAN/FR/1982/PHAROAH	CanL	ND/France	I	^(14)^
MHOM/TU/1980/IPT1	VL	ND/Tunisia	I	^(14)^
MCAN/AL/1983/LIPA116	CanL	ND/Algeria	I	^(14)^
MHOM/BR/74/PP75	VL	Icatu/BA/Brazil	II	^(13)^
MHOM/BR/70/BH46	VL	Conselheiro Pena/MG/Brazil	III	^(14)^
MHOM/HN/2017/AM-65	NUCL	Amapala, Honduras	I	--
MHOM/HN/2017/AM-73,	NUCL	Amapala, Honduras	I	--
MHOM/HN/2018/AMA-161	NUCL	Amapala, Honduras	I	--
MHOM/HN/2018/AMA-614	NUCL	Amapala, Honduras	I	--
*Leishmania donovani*				
MHOM/SD/00/1S-2D	VL	ND/Sudan	I	^(20)^
MHOM/IN/1983/Mongi-142	VL	ND/India	III	^(27)^

*a*: The World Health Organization (WHO) code is as follows: host (MHOM, *Homo sapiens*; MCAN, *Canis familiaris*)/country/year of isolation/name of strain; *b*: VL = visceral leishmaniasis, CanL = canine leishmaniasis, NUCL = non-ulcerated cutaneous leishmaniasis, ND = not determined; *c*: Brazilian states (MG = Minas Gerais, BA = Bahia, PB = Paraíba, MS = Mato Grosso do Sul, ES = Espírito Santo, SE = Sergipe.

Next, we evaluated the pro-inflammatory activity of the LPGs from the four dermotropic strains of *L. infantum* (AM-65, AM-73, AMA-161 and AMA-614) compared to viscerotropic strains (BH46 and BA262). Thioglycollate-elicited (Sigma) peritoneal macrophages were removed from C57BL/6 and BALB/c mice by peritoneal washing. Cells (3.5 X 10^5^ cells/well) were cultured in a sterile 96-well plate in Roswell Park Memorial Institute (RPMI) medium (Gibco, Waltham, MA) and primed with IFN-γ (100 IU/mL) (R&D Systems, Minneapolis, MN).^18^ Macrophages were exposed to lipopolysaccharide (LPS) (Sigma) (0.1 µg/mL - positive control); LPGs (10 µg/mL) and RPMI 1640 medium only (negative control). Culture supernatants were collected after 72 h and nitrite concentrations were determined by Griess reaction (Sigma). IL-6 and TNF-α were determined using BD CBA Mouse Cytokine assay kits according to the manufacturer’s specifications (BD Biosciences, CA, USA).^18^ In general, LPGs from all dermotropic strains were more pro-inflammatory than those from viscerotropic strains not only in BALB/c but also in C57BL/6 mice. In both mouse lineages, LPGs from dermotropic strains were comparable to LPS (positive control) or even higher (strain AMA-161) in their ability to induce NO, IL-6 and TNF-α (Fig. 2, Fig. 3A-B). Additionally, inner intraspecies variations were observed in the viscerotropic strains LPGs for both mice (BH46 versus BA262, p < 0.05). The low ability of BA262 LPG in inducing NO synthesis was already reported.^14^ Consistent with these observations, LPG1 knockouts of this strain induced higher levels of NO in RAW 264.7 cells confirming the role of LPG in this process.^24^ For dermotropic strains these differences were more evident for BALB/c (AMA-161 versus AM-65, AM-73 and AMA-614, p < 0.05). In C57BL/6, a higher NO production was detected for AMA-161, followed by AM-65 and AMA-73/AMA-614 (p < 0.05). Previous reports from our group^14^ showed that differences in LPG structures in *L. infantum* were determinant for NO production. This correlation was not easily demonstrated here, suggesting that perhaps intraspecies polymorphisms in the lipid anchors and/or the length of the repeat units motif of the dermotropic strains could also be responsible for higher NO and cytokine induction.^25^ ^,^ ^26^ Depending on the species and/or glycoconjugates, NO production was usually higher for C57BL/6 mice and did not show major variations for cytokines.^14^ ^,^ ^26^ However, these studies used a limited number of strains. Here, with an expanded panel of *L. infantum* strains, we decided to use both mice subsets for comparison. Interestingly, NO, IL-6 and TNF-α production were similar for both mice lineages. LPG from strain AMA-161 was observed even in levels higher than those of LPS (Figs 2 and 3, p < 0.05). Confirming our previous observations,^15^ NO and cytokine induction by BH46 LPG was very low in macrophages from BALB/c and C57BL/6 mice. Overall, macrophages activation by LPG from dermotropic species was much higher than those from the two viscerotropic strains (BA262 and BH46) (Figs 2 and 3, p < 0.05). These strains possess type I and type III LPGs, respectively. Since the LPGs from all Honduran strains are type I. As mentioned above, differences in stimulation could be not only a result of the length of the LPG but also the type of lipid anchor. LPG has four parts and several reports have shown that both glycan and lipid motifs are important for macrophage stimulation not only by LPGs but also for glycoinositolphospholipids (GIPLs).^25^ ^,^ ^26^ *In vitro* experiments with dermotropic strains did not show evident differences in their ability to infect and survive in hamster peritoneal macrophages (MD Laurenti, Unpublished observations). This strain was isolated from an older patient (69 years), whose lesion had a longer evolution time (3 years), which could be reflecting a more balanced parasite-host adaptation. This higher NO stimulation by LPGs from dermotropic species, together with TGF-β, may explain low parasite loads observed in those lesions.^9^ This was very surprising since LPGs from viscerotropic species (*L. infantum* and *L. donovani* are usually more immunosuppressive.^15^ ^,^ ^27^ ^,^ ^28^ ^,^ ^29^ This suggests that the activity of LPGs from dermotropic *L. infantum* resembles to that of *L. braziliensis*, *L. enriettii* and *L. amazonensis*.^15^ ^,^ ^18^ ^,^ ^23^ ^,^ ^30^ This reinforces that dermotropic *L. infantum* with respect to these mediators (NO and cytokines) are behaving very similar to cutaneous species.

**Fig. 2:**
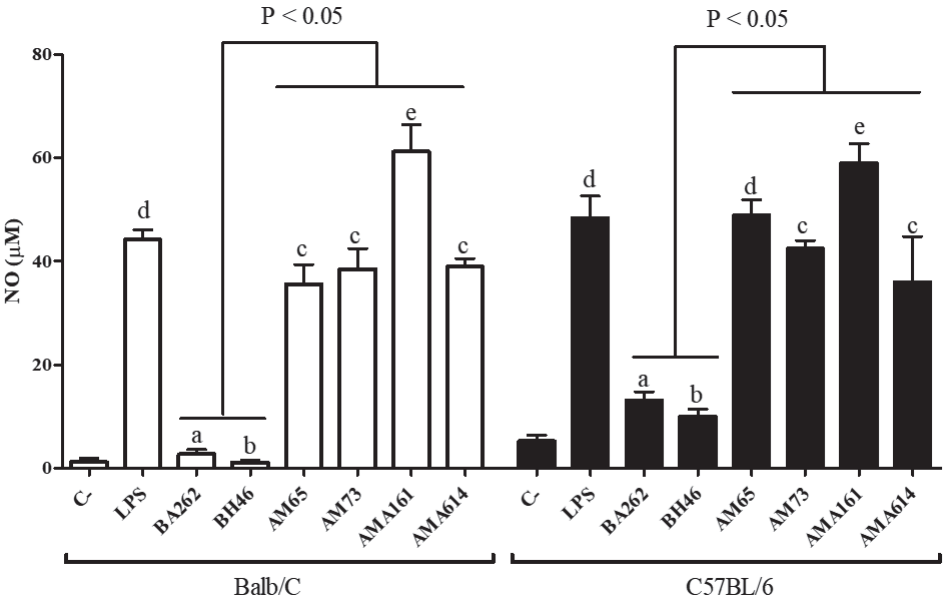
nitric oxide (NO) production by murine peritoneal macrophages (BALB⁄c and C57BL/6) exposed to lipophosphoglycan (LPG) (10 µg/mL) from dermotropic (AM65, AM73, AMA161 and AMA614) and viscerotropic strains (BA262 and BH46) of *Leishmania infantum*. Lipopolysaccharide (LPS) (0.1 µg/mL) was used as positive control. Results were expressed as the mean ± standard deviation of three independent experiments. Statistical analysis was achieved using the nonparametric Kruskal-Wallis test followed by Dunn’s post hoc test for multiple comparisons among groups (lines above bars). T-Student´s t test was used to compare each sample and letters above bars indicate statistical differences (p < 0.05).

**Fig. 3:**
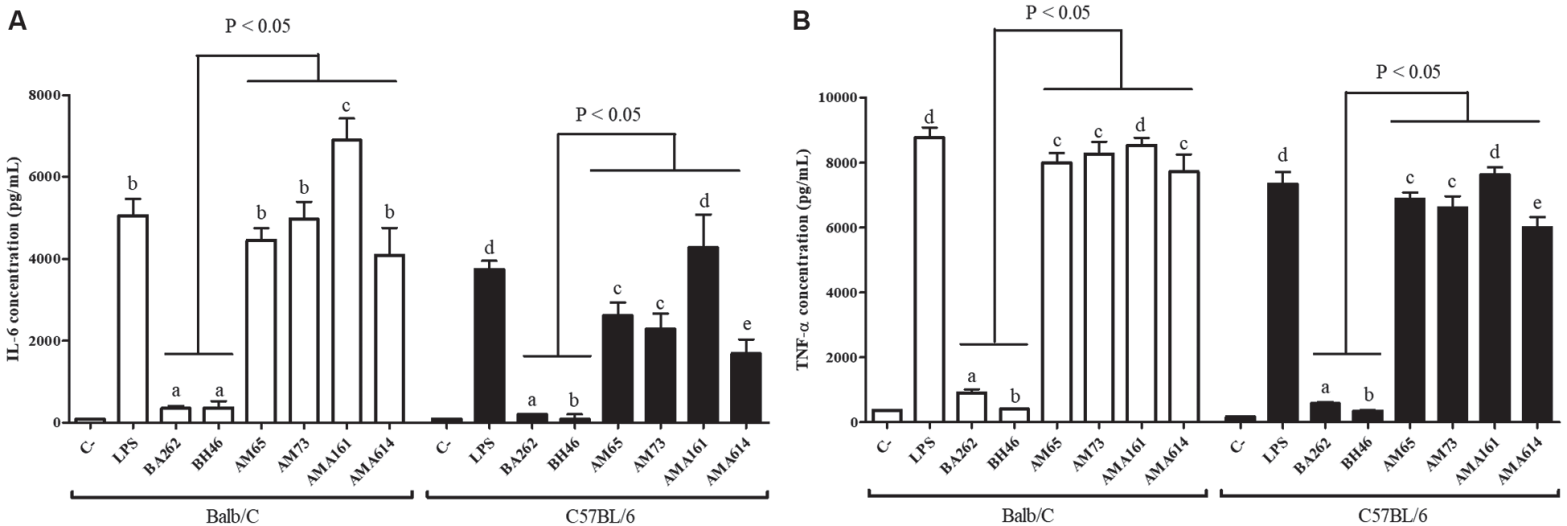
IL-6 (A) and TNF-α (B) production by murine peritoneal macrophages (BALB/c and C57BL/6) exposed to lipophosphoglycan (LPG) (10 µg/mL) from dermotropic (AM65, AM73, AMA161 and AMA614) and viscerotropic strains (BA262 and BH46) of *Leishmania infantum*. Lipopolysaccharide (LPS) (0.1 µg/mL) was used as positive control. Results were expressed as the mean ± standard deviation of three independent experiments. Statistical analysis was achieved using the nonparametric Kruskal-Wallis test followed by Dunn’s post hoc test for multiple comparisons among groups (lines above bars). T-Student´s t test was used to compare each sample and letters above bars indicate statistical differences (p < 0.05).

In conclusion, *L. infantum* has been shown to cause a wide spectrum of manifestations, from benign skin lesions to fatal visceral forms. In this work, we characterised the structure of the LPG from four NUCL strains. Despite the LPGs having a type I structure, they triggered higher NO and cytokine production than those from viscerotropic strains, a pattern often observed in other cutaneous *Leishmania* species.

*Ethics statement* - All animals were handled in strict accordance with animal practice as defined by the Internal Ethics Committee in Animal Experimentation (CEUA) of Fundação Oswaldo Cruz (FIOCRUZ), Belo Horizonte, Minas Gerais (MG), Brazil (protocol P-17/14-2). This protocol followed the guidelines of CONCEA/MCT.
